# Extensive next-generation sequencing analysis in chronic lymphocytic leukemia at diagnosis: clinical and biological correlations

**DOI:** 10.1186/s13045-016-0320-z

**Published:** 2016-09-15

**Authors:** Gian Matteo Rigolin, Elena Saccenti, Cristian Bassi, Laura Lupini, Francesca Maria Quaglia, Maurizio Cavallari, Sara Martinelli, Luca Formigaro, Enrico Lista, Maria Antonella Bardi, Eleonora Volta, Elisa Tammiso, Aurora Melandri, Antonio Urso, Francesco Cavazzini, Massimo Negrini, Antonio Cuneo

**Affiliations:** 1 Hematology Section, Department of Medical Sciences, Azienda Ospedaliero-Universitaria, Arcispedale S. Anna, University of Ferrara, Via Aldo Moro, 8, 44124 Ferrara, Cona Italy; 2 Department of Morphology, Surgery and Experimental Medicine, and “Laboratorio per le Tecnologie delle Terapie Avanzate” (LTTA), University of Ferrara, Ferrara, Italy

**Keywords:** Chronic lymphocytic leukemia, Gene mutation analysis, Next-generation sequencing, Complex karyotype, Prognosis

## Abstract

**Background:**

In chronic lymphocytic leukemia (CLL), next-generation sequencing (NGS) analysis represents a sensitive, reproducible, and resource-efficient technique for routine screening of gene mutations.

**Methods:**

We performed an extensive biologic characterization of newly diagnosed CLL, including NGS analysis of 20 genes frequently mutated in CLL and karyotype analysis to assess whether NGS and karyotype results could be of clinical relevance in the refinement of prognosis and assessment of risk of progression. The genomic DNA from peripheral blood samples of 200 consecutive CLL patients was analyzed using Ion Torrent Personal Genome Machine, a NGS platform that uses semiconductor sequencing technology. Karyotype analysis was performed using efficient mitogens.

**Results:**

Mutations were detected in 42.0 % of cases with 42.8 % of mutated patients presenting 2 or more mutations. The presence of mutations by NGS was associated with unmutated *IGHV* gene (*p* = 0.009), CD38 positivity (*p* = 0.010), risk stratification by fluorescence in situ hybridization (FISH) (*p* < 0.001), and the complex karyotype (*p* = 0.003). A high risk as assessed by FISH analysis was associated with mutations affecting *TP53* (*p* = 0.012), *BIRC3* (*p* = 0.003), and *FBXW7* (*p* = 0.003) while the complex karyotype was significantly associated with *TP53*, *ATM*, and *MYD88* mutations (*p* = 0.003, 0.018, and 0.001, respectively). By multivariate analysis, the multi-hit profile (≥2 mutations by NGS) was independently associated with a shorter time to first treatment (*p* = 0.004) along with *TP53* disruption (*p* = 0.040), *IGHV* unmutated status (*p* < 0.001), and advanced stage (*p* < 0.001). Advanced stage (*p* = 0.010), *TP53* disruption (*p* < 0.001), *IGHV* unmutated status (*p* = 0.020), and the complex karyotype (*p* = 0.007) were independently associated with a shorter overall survival.

**Conclusions:**

At diagnosis, an extensive biologic characterization including NGS and karyotype analyses using novel mitogens may offer new perspectives for a better refinement of risk stratification that could be of help in the clinical management of CLL patients.

**Electronic supplementary material:**

The online version of this article (doi:10.1186/s13045-016-0320-z) contains supplementary material, which is available to authorized users.

## Background

Chronic lymphocytic leukemia (CLL) displays a heterogeneous clinical course [1–3], some patients living for years with asymptomatic disease and others experiencing early progression requiring therapeutic intervention. Modern treatment algorithms must take into account age, comorbidities, and prognostic/predictive factors, including genetic lesions [4]. Adverse prognostic factors include stage [5], positivity for CD38, ZAP70, and CD49d [6–8], and, among genetic features, the unmutated configuration of the variable region of the immunoglobulin heavy chain gene (*IGHV*) [6] and specific molecular cytogenetic lesions revealed by fluorescent in situ hybridization (FISH). More recently, karyotype aberrations were shown to represent strong prognostic factors [9–14], and large retrospective studies demonstrated that *TP53*, *NOTCH1*, and *SF3B1* gene mutations have a negative impact on the time to first treatment (TTFT) and overall survival (OS) [15–17]. These data were in part confirmed by prospective clinical trials using homogeneous treatment protocols [18, 19], and recurrent genomic lesions were included within comprehensive prognostic indexes [20, 21] helping clinicians to counsel patients more appropriately, to define the follow-up interval, and, potentially, to provide a rational basis to design early intervention protocols for high-risk patients [22].

Next-generation sequencing (NGS) techniques documented that, besides the aforementioned genes, a number of previously unidentified genes may be mutated in CLL and that the disruption of putative core cellular pathways represents an important mechanism promoting disease progression and drug resistance [23–26]. NGS may detect minor cell populations (subclones) harboring a variety of gene mutations, including *NOTCH1*, *SF3B1*, *BIRC3*, and *TP53* mutations, the latter having a negative prognostic impact that was similar to TP53 clonal mutations [27–29] as detected by conventional sequencing techniques (i.e., Sanger sequencing).

Thus, NGS is becoming of age for usage in clinical practice, and indeed, over 50 % of CLL patients were shown to carry mutations in one or more genes [30, 31], potentially making NGS a sensitive tool for the detection of mutations including subclonal mutations.

To assess whether an extended mutational screening by NGS at diagnosis could allow for a refinement of our capability to predict TTFT and OS, we designed a CLL-specific gene panel, covering hotspots or complete coding regions of 20 genes more frequently mutated in CLL. We performed NGS of these 20 genes using a resource-efficient platform in 200 consecutive newly diagnosed patients representing over 90 % of CLL incident cases in our region. By correlating mutational data obtained by an extensive genetic/cytogenetic characterization with clinic-biological parameters and outcome, we were able to show that NGS screening was an independent prognostic factor for TTFT and that complex karyotype was a strong predictor of an inferior survival in this patient population.

## Methods

### Patients

The study cohort consisted of 200 consecutive untreated CLL patients diagnosed and followed between 2007 and 2014. All patients were diagnosed according to NCI criteria [32]. Only patients with a Matutes immunophenotypic score [33] ≥3 (i.e., typical CLL) were included. CD38 and ZAP-70 were tested on peripheral blood (PB) cells, as described [34]. When needed, mantle cell lymphoma was excluded by the evaluation of cyclin D1. The study was approved by the local ethics committee. Indications for treatment included increased white blood cell count with <6 month lymphocyte doubling time, anemia or thrombocytopenia due to bone marrow infiltration or autoimmune phenomena not responding to steroids, and disease progression in the Binet staging system. Fludarabine and bendamustine (since 2010), containing regimens in association with or without rituximab, were used as first-line treatment; chlorambucil was used in elderly and unfit patients according to shared treatment policy adopted at our center.

### Cytogenetic and FISH analyses

Interphase FISH was performed on PB samples obtained at diagnosis using probes for the following regions: 13q14, 12q13, 11q22/ATM, and 17p13/TP53 (Vysis/Abbott Co, Downers Grove, IL) as described [35]. Each patient was categorized into a FISH risk group according to the following classification: favorable group (isolated 13q14 deletion or absence of FISH aberrations), unfavorable group (deletions of 11q22 or of 17p13), and intermediate group (trisomy 12).

Cytogenetic analysis was performed on the same samples used for FISH analysis using CpG-oligonucleotide DSP30 (2 μmol/l TibMolBiol Berlin, Germany) plus IL2 (100 U/ml Stem Cell Technologies Inc., Milan, Italy) as described [36]. The complex karyotype was defined by the presence of at least 3 chromosome aberrations.

### *IGHV* analysis

*IGHV* genes were amplified from genomic DNA and sequenced according to standard methods with the cutoff of 98 % homology to the germline sequence to discriminate between mutated (<98 %) and unmutated (≥98 %) cases, as reported [35].

### Ion Torrent Personal Genome Machine (PGM) analysis

NGS analysis was performed on the same samples used for FISH and cytogenetic analyses. In all samples, the percentage of CLL cells was over 90 % as assessed by flow cytometry analysis. Agilent HaloPlex Target Enrichment kit (Agilent Technologies, Santa Clara, CA, USA) was used to produce libraries of exonic regions from 20 genes (*ATM*, *BIRC3*, *BRAF*, *CDKN2A*, *PTEN*, *CDH2*, *DDX3X*, *FBXW7*, *KIT*, *KLHL6*, *KRAS*, *MYD88*, *NOTCH1*, *NRAS*, *PIK3CA*, *POT1*, *SF3B1*, *TP53*, *XPO1*, *ZMYM3*) starting from genomic DNA from PB samples, according to HaloPlex Target Enrichment System (Agilent Technologies, Santa Clara, CA, USA). Diluted libraries were linked to Ion Sphere Particles, clonally amplified in an emulsion PCR and enriched using Ion OneTouch emulsion PCR System (Life technologies, Foster City, CA, USA). Exon-enriched DNA was precipitated with magnetic beads coated with streptavidin. Enriched, template-positive Ion Sphere Particles were loaded in one ion chip and sequenced using Ion Torrent PGM (Life technologies, Foster City, CA, USA). Sequencing data were aligned to the human reference genome (GRCh37). Data analysis and variant identification were performed using Torrent Suite 3.4 and Variant Caller plugin 3.4.4 (Life technologies, Foster City, CA, USA) [37].

### Statistical analysis

The Mann-Whitney and the Pearson’s chi-squared tests were applied for quantitative and categorical variables, respectively. TTFT was calculated as the interval between diagnosis and the start of first-line treatment. OS was calculated from the date of diagnosis until death due to any cause or until the last patient follow-up. Survival curves were compared by the log-rank test. Proportional hazards regression analysis was used to identify the significant independent prognostic variables on TTFT. The stability of the Cox model was internally validated using bootstrapping procedures [15]. Statistical analysis was performed using Stata 14.0 (Stata Corp, College Station, TX).

## Results

### Patients and mutation analyses of the 20 genes by NGS

The clinical and biologic characteristics of the 200 CLL patients are presented in Table 1.

**Table 1:** Clinical and biological characteristics of the 200 CLL patients

Variable	
Age, median yrs (range)	67.6 (38.3–89.9)
Sex m/f	121/79
Binet stage a/b/c	161/25/14
CD38 neg/pos	121/79
ZAP-70 neg/pos	143/37
*IGVH* mut/unmut	105/91
13q14 deletion yes/no	104/96
Trisomy 12 yes/no	32/168
11q22 deletion yes/no	20/180
17p13 deletion yes/no	9/191
FISH fav/int/unfav	142/30/28
Complex karyotype no/yes	167/28
Mutated patients by NGS no/yes	116/84
No. of mutations by NGS 0/1/2/3/4	116/48/24/8/4
*TP53* mut/WT	16/184
*TP53* disruption yes/no	19/181

*f* female, *fav* favorable, *int* intermediate, *m* male, *mut* mutated, *neg* negative, *pos* positive, *unfav* unfavorable, *unmut* unmutated, *yrs* years, *TP53 disruption* 17p13 deletion and/or TP53 mutation

Parallel sequencing of exonic regions from the 20 genes showed somatic mutations in 84/200 (42.0 %) cases. One hundred thirty-six mutations were found in these 84 patients; 114 missense mutations, 7 nonsense mutations, 14 frameshit deletions, and 1 frameshit insertion. Mutations were detected with a frequency ranging from 5.0 to 96.7 % of the reads. Sixteen cases (8.0 %) showed mutations in the *TP53* gene, 16 (8.0 %) in the *NOTCH1* gene, 15 (7.5 %) in the *SF3B1* gene, 10 (5.0 %) in the *ATM* gene, 8 (4.0 %) in the *BIRC3* gene, 7 (3.5 %) in the *MYD88* gene, 7 (3.5 %) in the *PTEN* gene, 6 (3.0 %) in the *FBXW7* gene, 5 (2.5 %) in the *POT1* gene, 5 (2.5 %) in the *BRAF* gene, 5 (2.5 %) in the *ZMYM3* gene, and 19 (9.5 %) cases in the remaining 9 genes (Additional file 1: Table S1). 36/84 (42.8 %) mutated patients presented 2 or more mutations (Additional file 2: Table S2). *TP53* mutations (*p* = 0.027) were significantly more frequent among patients with 2 or more mutations while a trend was observed for *BIRC3* mutations (*p* = 0.059) and mutations of genes less frequently mutated in CLL (*p* = 0.057) (Additional file 3: Table S3).

### Correlations between mutational status by NGS, molecular cytogenetic findings, and clinico-biological parameters

The presence of somatic mutations did not correlate with sex, age, and Binet stage while the occurrence of mutations by NGS analysis was significantly associated with CD38 positivity (*p* = 0.010), *IGHV* unmutated status (*p* = 0.009), intermediate high-risk cytogenetics by FISH analysis (*p* < 0.001), and the complex karyotype (*p* = 0.003; Table 2).

**Table 2:** Correlations between mutational status by NGS analysis and clinical biological parameters

	Mutated (*n* = 84)	Not mutated (*n* = 116)	*p*
Sex m/f	49/35	72/44	0.594
Age <70/≥70 years	46/38	69/47	0.505
Binet stage a/b/c	66/12/6	95/13/8	0.802
CD38 neg/pos	42/42	79/37	0.010
*IGHV* mut/unmut	36/48	69/43	0.009
FISH fav/int unfav	48/36	94/22	<0.001
Complex karyotype no/yes	63/19	104/9	0.003

*f* female, *fav* favorable, *int* intermediate, *m* male, *mut* mutated, *neg* negative, *pos* positive, *unfav* unfavorable, *unmut* unmutated, *yrs* years, *TP53 disruption* 17p13 deletion and/or TP53 mutation

**Fig. 1:**
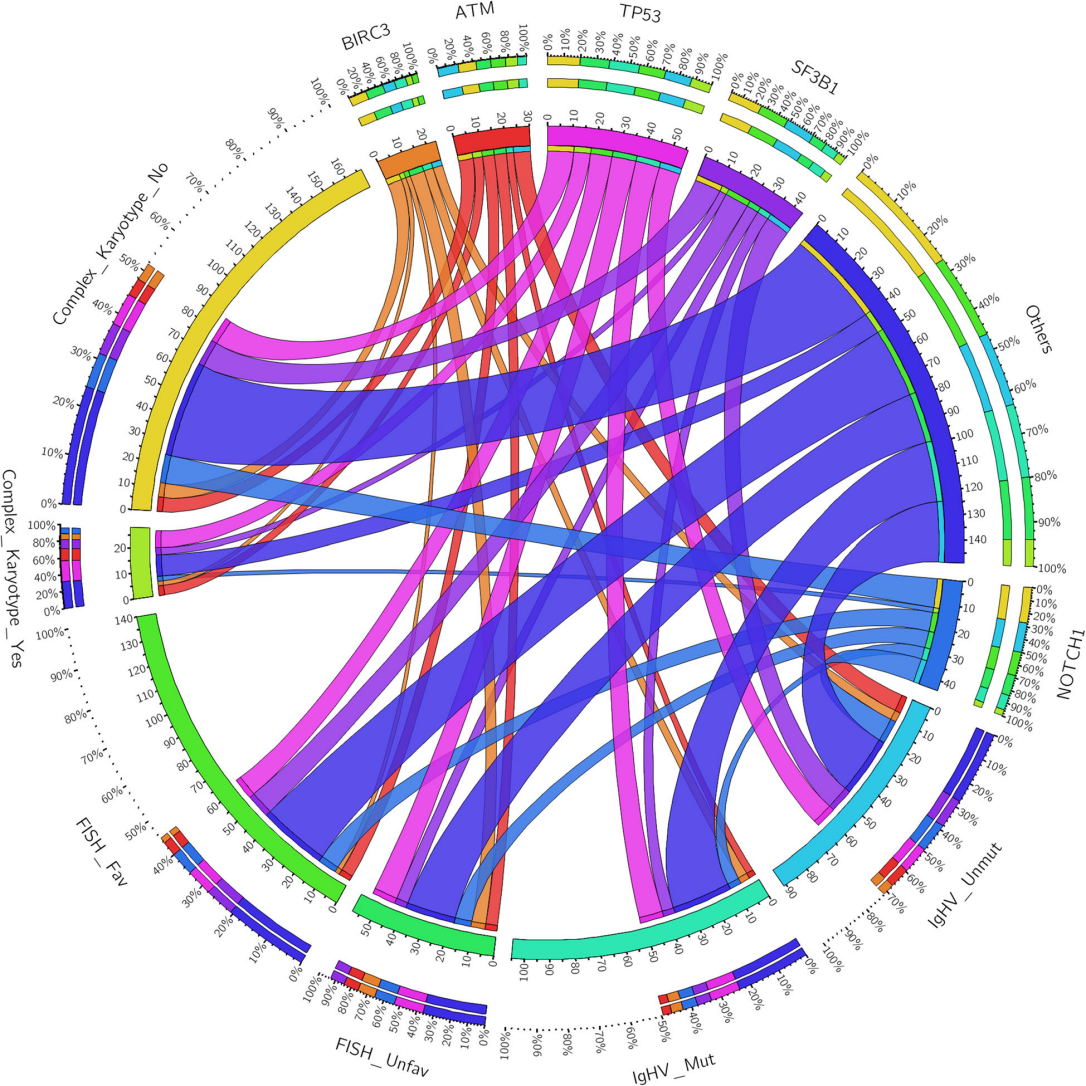
Gene mutations and correlation with genomic features: circos diagrams illustrating pairwise co-occurrence of gene mutations with *IGHV* status, FISH results, and complex karyotype

A higher risk as assessed by FISH analysis was associated with the presence of mutations affecting *TP53* (*p* = 0.012), *BIRC3* (*p* = 0.003), and *FBXW7* (*p* = 0.003) while the complex karyotype was significantly associated with *TP53*, *ATM*, and *MYD88* mutations (*p* = 0.003, 0.018, and 0.001, respectively: Table 3; Fig. 1).

**Table 3:** Correlations between mutations by NGS analysis, FISH results, and karyotype complexity

	FISH results			Complex karyotype		
	Fav	Int-unfav		No	Yes	*p*
No. of mutations by NGS no/1/≥2	94/28/20	22/19/17	0.001	104/36/27	9/11/8	0.011
*TP53* WT/mut	135/7	49/9	0.012	158/9	22/6	0.003
*NOTCH1* WT/mut	133/9	51/7	0.175	155/12	25/3	0.517
*SF3B1* WT/mut	132/10	53/5	0.701	156/11	25/3	0.434
*ATM* WT/mut	137/5	53/5	0.133	161/6	24/4	0.018
*BIRC3* WT/mut	140/2	52/6	0.003	161/6	26/2	0.381
*MYD88* WT/mut	136/6	57/1	0.382	164/3	24/4	0.001
*PTEN* WT/mut	138/4	55/3	0.411	161/6	27/1	0.996
*FBXW7* WT/mut	141/1	53/5	0.003	161/6	28/0	0.308
*POT1* WT/mut	138/4	57/1	0.653	162/5	28/0	0.354
*BRAF* WT/mut	139/3	56/2	0.583	163/4	28/0	0.408
*ZMYM3* WT/mut	138/4	57/1	0.653	163/4	27/1	0.716
Others WT/mut	129/13	49/6	0.192	149/18	24/4	0.587

*f* female, *fav* favorable, *int* intermediate, *m* male, *mut* mutated, *neg* negative, *pos* positive, *unfav* unfavorable, *unmut* unmutated

**Fig. 2:**
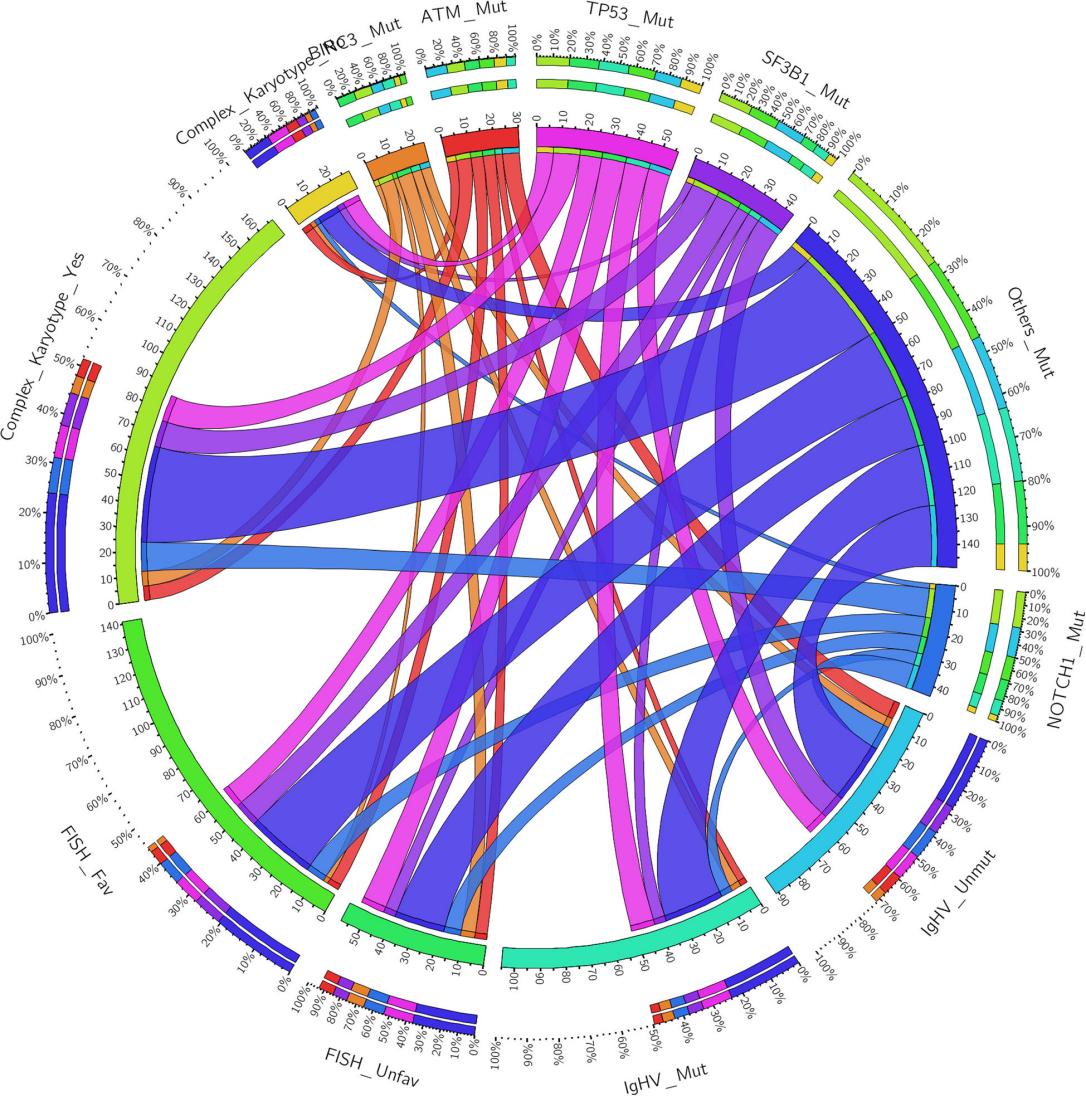
TTFT according to number of mutations by NGS analysis (*p* < 0.001)

The median follow-up for the 200 CLL patients was 52.3 months. In univariate analysis (Table 4), the occurrence of mutations and the presence of 2 or more mutations were significantly associated with a worse TTFT (Fig. 2) along with advanced Binet stage; CD38 positivity; *IGHV* unmutated status; intermediate unfavorable FISH results; 11q22 deletion, 17p13 deletion, and/or TP53 mutations (here referred to as *TP53* disruption); and complex karyotype. A shorter TTFT was also observed for *TP53-*, *NOTCH1-*, *ATM-*, and *BRAF-*mutated patients. By multivariate analysis (Table 5), we found that the multi-hit profile (≥2 mutations by NGS) predicted a shorter TTFT (*p* = 0.004) along with TP53 disruption (*p* = 0.040), *IGHV* unmutated status (*p* < 0.001), and advanced stage (*p* < 0.001).

**Table 4:** Univariate analysis for TTFT and OS

		TTFT		OS	
Variable	*N* pts	HR (CI 95 %)	*p*	HR (CI 95 %)	*p*
Binet stage B–C vs A	39 vs 161	9.884 (5.939–16.450)	<0.0001	3.174 (1.677–6.007)	0.0002
CD38 pos vs neg	79 vs 121	4.097 (2.564–6.546)	<0.0001	3.123 (1.686–5.783)	0.0001
IGVH mut vs unmut	105 vs 91	5.584 (3.326–9.374)	<0.0001	3.667 (1.886–7.127)	<0.0001
11q22 deletion yes vs no	20 vs 180	2.879 (1.528–5.426)	0.0006	1.736 (0.739–4.078)	0.2000
*TP53* disruption yes/no	19 vs 181	3.284 (1.867–5.781)	<0.0001	4.246 (2.076–8.687)	<0.0001
FISH int-unfav vs fav	58 vs 142	2.605 (1.670–4.063)	<0.0001	2.432 (1.438–4.454)	0.0029
Complex karyotype yes vs no	28 vs 167	2.979 (1.756–5.056)	<0.0001	3.854 (1.961–7.578)	<0.0001
Mutations by NGS no/yes	116 vs 84	2.835 (1.799–4.469)	<0.0001	2.171 (1.176–4.008)	0.0130
Number of mutations by NGS					
0	116	1	<0.001	1	0.037
1	47	2.373 (1.369–4.112)	0.002^a^	1.936 (0.930–4.032)	0.078^a^
≥2	37	3.418 (2.009–5.759)	<0.001^a^	2.466 (1.187–5.126)	0.016^a^
*TP53* mut vs wt	16 vs 184	2.804 (1.514–5.194)	0.0010	2.793 (1.284–6.098)	0.0069
*NOTCH1* mut vs wt	16 vs 184	2.353 (1.164–4.762)	0.0141	2.646 (1.114–6.259)	0.0219
*SF3B1* mut vs wt	15 vs 185	1.779 (0.886–3.571)	0.1006	1.170 (0.419–3.268)	0.7648
*ATM* mut vs wt	10 vs 190	3.623 (1.715–7.633)	0.0003	1.946 (0.686–5.525)	0.2023
*BIRC3* mut vs wt	8 vs 192	0.817 (0.254–2.597)	0.7246	1.099 (0.252–4.808)	0.8998
*MYD88* mut vs WT	7 vs 193	1.758 (0.642–4.812)	0.2724	1.505 (0.363–6.240)	0.5733
*PTEN* mut vs WT	7 vs 193	1.573 (0.574–4.310)	0.3780	1.503 (0.363–6.224)	0.5742
*FBXW7* mut vs WT	6 vs 194	1.820 (0.664–4.988)	0.2441	1.445 (0.349–5.986)	0.6134
*POT1* mut vs WT	5 vs 195	1.059 (0.259–0.321)	0.9375	0.978 (0.352–4.768)	0.9973
*BRAF* mut vs WT	5 vs 195	7.730 (3.014–19.827)	<0.0001	2.126 (0.286–15.823)	0.4610
*ZMYM3* mut vs WT	5 vs 195	0.484 (0.067–3.480)	0.4710	2.336 (0.563–9.693)	0.2434
*OTHERS mut* vs *wt*	19 vs 181	1.036 (0.517–2.075)	0.9205	0.898 (0.320–2.518)	0.8381

^a^Compared with no mutation

*f* female, *fav* favorable, *int* intermediate, *m* male, *mut* mutated, *neg* negative, *pos* positive, *unfav* unfavorable, *unmut* unmutated, *yrs* years, *TP53 disruption* 17p13 deletion and/or TP53 mutation

**Table 5:** Multivariate analysis for TTFT and OS

			TTFT					OS		
				After bootstrapping					After bootstrapping	
Variable	HR	CI	*p*	CI	*p*	HR	CI	*p*	CI	*p*
Binet stage b–c vs a	11.206	6.384–19.671	<0.001	5.570–22.545	<0.001	3.080	1.501–6.319	0.002	1.302–7.286	0.010
CD38 pos vs neg	1.141	0.670–1.942	0.627	0.663–1.938	0.634	1.067	0.506–2.249	0.864	0.448–2.356	0.883
11q deletion yes vs no	1.306	0.619–2.755	0.484	0.532–3.205	0.560	Na	Na	Na	Na	Na
*TP53* disruption yes vs no	2.255	1.168–4.352	0.015	1.039–4.891	0.040	4.055	1.844–7.917	<0.001	1.897–8.670	<0.001
*IGHV* unmut vs mut	5.078	2.599–9.554	<0.001	2.491–10.354	<0.001	3.198	1.524–6.13	0.002	1.200–8.522	0.020
No. of mutations by NGS										
0	1			1		1			1	
1	1.452	0.812–2.594	0.208	0.574–3.673	0.431	0.930	0.417–2.074	0.860	0.348–2.484	0.885
≥2	2.791	1.468–5.306	0.002	1.375–5.665	0.004	1.115	0.492–2.523	0.795	0.480–2.589	0.801
Complex karyotype yes vs no	1.649	0.896–3.034	0.108	0.824–3.301	0.158	3.173	1.521–6.619	0.002	1.369–7.355	0.007

*f* female, *fav* favorable, *int* intermediate, *m* male, *mut* mutated, *neg* negative, *pos* positive, *unfav* unfavorable, *unmut* unmutated, *yrs* years, *TP53 disruption* 17p13 deletion and/or TP53 mutation

When considering OS (Table 4), a poorer prognosis was associated with the occurrence of mutations by NGS analysis, the presence of 2 or more mutations, with *TP53* mutations, and with advanced stage, CD38 positivity, *IGHV* unmutated status, *TP53* disruption, and complex karyotype. In multivariate analysis, advanced stage (*p* = 0.010), *IGHV* unmutated status (*p* = 0.020), *TP53* disruption (*p* < 0.001), and the complex karyotype (*p* = 0.007) independently predicted a worse outcome (Table 5).

## Discussion

CLL is the most frequent leukemia in western countries and has a significant socioeconomic impact. It is therefore important to define which patients are at higher risk of progression and therefore require stricter follow-up and which genetic lesions are associated with risk of relapse and/or chemorefractoriness ultimately determining a shorter survival [22]. Unlike previous reports analyzing prognostic/predictive factors in CLL requiring treatment at the time of progression, we were able to perform an extensive biologic characterization in an unselected prospective series of 200 patients diagnosed over an 8-year span and followed for a median of 52.3 months over the last 10 years. Our center has a >90 % capture of each incident case of CLL in our region of approximately 400,000 inhabitants because the diagnosis of CLL in our province was centralized since 2006. With the exception of frail patients with a significant number of comorbidities precluding any form of specific treatment, whom were not submitted to extensive molecular cytogenetic characterization, the patient population included in this analysis is highly representative of the true nature of CLL and allows meaningful analyses of TTFT and OS in a real-world scenario.

The Ion Torrent PGM is a NGS platform that uses semiconductor sequencing technology. In clinical practice, PGM may represent a very sensitive tool for mutational screening of patients with CLL, allowing multiplexing of samples and gene targets in one experimental setup [30] and resulting in higher speed of analysis and lower costs [38]. Parallel sequencing of exonic regions in these 20 CLL-related genes showed somatic mutations in 84/200 (42.0 %) cases by using a 5 % cutoff. Mutations were detected with a frequency ranging from 5.0 to 96.7 % of the reads, clearly showing that both major and minor clonal mutations were present, the former representing early leukemogenetic events and the latter representing late-appearing aberrations possibly associated with disease progression or chemorefractoriness [39, 40].

In this series, the frequency of mutations involving *TP53*, *NOTCH1*, *SF3B1*, *ATM*, and *BIRC3* genes clearly reflects the nature of our patient cohort that included untreated CLL analyzed early during the natural history of the disease and comprising 80.5 % of Binet stage A cases. Approximately, the same incidence for these mutations was reported in a series of CLL patients observed in the general practice and not enrolled in clinical trials [17]. The frequency of mutations involving the other investigated genes was in line with data published in literature using whole exome sequencing [41–44].

Interestingly, we observed that 18.0 % of the cases presented more than one mutation. In the CLL11 trial, 161 patients were evaluated at the time of treatment requirement and NGS analysis revealed mutations in 42 out of 85 analyzed genes, with 76.4 and 42.2 % of the patients presenting at least one or ≥2 genes affected by mutations, respectively [14].

In our series of patients, the occurrence of mutations was associated with adverse molecular and genetic findings including *IGVH* unmutated status, intermediate high-risk FISH results, and the presence of a complex karyotype. Noteworthy, a higher incidence of concurrent mutations was observed in *TP53*-mutated patients, while the presence of a complex karyotype was associated with *TP53-*, *ATM-*, and *MYD88-*mutated cases. These results suggest that concurrent mutations, as well as complex karyotype, might represent an aspect of genetic instability correlated to a defective DNA damage response [45].

We then analyzed the correlation between the mutational status and outcome. A shorter TTFT was observed in those patients with mutations by NGS and with mutations involving *TP53*, *NOTCH1*, *ATM*, and *BRAF*. The prognostic significance of BRAF mutations needs to be confirmed on larger series because it was derived from a limited number of patients, most of whom had concurrent mutations of other genes. By multivariate analysis, we found that the multi-hit profile (≥2 mutations by NGS) was independently associated with a shorter TTFT along with *TP53* disruption, *IGHV* unmutated status, and advanced stage.

Given the complexity of CLL genetic landscape, we suggest that not only the presence of clones or subclones [46] but also the concurrent presence of mutations may play a significant role in prognostication. This study, to our knowledge, provides the first demonstration that at diagnosis, in an unselected CLL patient population followed up at one center having a >90 % capture of incident cases, a multi-hit profile derived from an extensive NGS analysis is independently associated with a shorter TTFT. Noteworthy, concurrent gene mutations are also frequent in patients with relapsed/refractory CLL and are associated with a worse outcome [47].

When considering OS, a poorer outcome was associated with the presence of mutations by NGS, with mutations in *TP53* and *NOTCH1* genes, with the multi-hit profile, with *IGHV* unmutated status, with *TP53* disruption, and with the complex karyotype. However, by multivariate analysis, only *TP53* disruption was independently associated with a worse outcome along with advanced stage, *IGHV* unmutated status, and the complex karyotype.

Whereas the strong independent impact on TTFT and OS of *IGHV* mutational status and *TP53* disruption was previously demonstrated [12, 14, 15, 45, 46], the finding of an independent impact on OS of the complex karyotype is noteworthy, especially when considering that an extensive clinic-biologic characterization was performed in this patient cohort. Recently, an independent prognostic relevance on OS of the complex karyotype has emerged in CLL patients investigated at different phases of the disease: at diagnosis [13, 34], before first-line treatment [14], and in refractory relapsed patients treated with ibrutinib [48]. We may assume that the complex karyotype probably reflects a high level of genomic instability that appears to be a better predictor of worse OS in comparison to single and multiple concurrent mutations, with the only exception of TP53 mutations. Thus, karyotyping seems to substantially contribute to the identification of CLL patients with most adverse prognosis and should be considered in an extensive diagnostic work-up in future CLL trials [49, 50].

## Conclusions

Altogether, our data suggests that NGS may play an important role in the definition of the risk of disease progression and therefore could be useful in the diagnostic work-up of CLL patients as an efficient, sensitive, and affordable technique for routine screening of mutations. Indeed, NGS analysis, in combination with clinical stage, *TP53* disruption, and *IGHV* assessment, may identify those patients that are at higher risk of progression and therefore need a stricter follow-up whereas karyotyping could represent along with *TP53* disruption the best genetic predictor of OS. However, some issues need to be better defined before the introduction of the extensive NGS approach into the routine clinical practice: (i) which genes and how many genes should be included in the work-up panel for an efficient and affordable routine applicability, (ii) what cutoff for mutational analysis should be considered clinically relevant, and (iii) how to develop a standardized methodology ensuring reproducibility of the results [51].

## Additional files

Additional file 1: Table S1. List of the mutations observed by NGS in the 84 mutated patients. (XLSX 22 kb) Additional file 2: Table S2. Overview of the mutations observed in the 20 genes. (DOCX 13 kb) Additional file 3: Table S3. Gene mutations according to the presence of 1 or ≥2 mutations by NGS analysis. (DOC 33 kb)

## Abbreviations

CLL: Chronic lymphocytic leukemia
FISH: Fluorescent in situ hybridization
IGHV: Immunoglobulin heavy chain gene
NGS: Next-generation sequencing
OS: Overall survival
PB: Peripheral blood
PGM: Personal Genome Machine
TTFT: Time to first treatment
