# Erratum to: Extensive next-generation sequencing analysis in chronic lymphocytic leukemia at diagnosis: clinical and biological correlations

**DOI:** 10.1186/s13045-016-0331-9

**Published:** 2016-09-30

**Authors:** Gian Matteo Rigolin, Elena Saccenti, Cristian Bassi, Laura Lupini, Francesca Maria Quaglia, Maurizio Cavallari, Sara Martinelli, Luca Formigaro, Enrico Lista, Maria Antonella Bardi, Eleonora Volta, Elisa Tammiso, Aurora Melandri, Antonio Urso, Francesco Cavazzini, Massimo Negrini, Antonio Cuneo

**Affiliations:** 1 Hematology Section, Department of Medical Sciences, Azienda Ospedaliero-Universitaria, Arcispedale S. Anna, University of Ferrara, Via Aldo Moro, 8, 44124 Ferrara, Cona, Italy; 2 Department of Morphology, Surgery and Experimental Medicine, and “Laboratorio per le Tecnologie delle Terapie Avanzate” (LTTA), University of Ferrara, Ferrara, Italy

## Erratum

*n.b. The error described below was mistakenly carried forward by the production team handling this article, and thus was****not****the fault of the authors*.

The original version of this article [1] had a duplication of Fig. 1 in place of where Fig. 2 should have been, resulting in two displays of Fig. 1 and the absence of Fig. 2.

The article has now been updated to remove the duplicate of Fig. 1 and to insert the correct Fig. 2 into its appropriate place.

The two figures in question can be seen below.

**Fig. 1:**
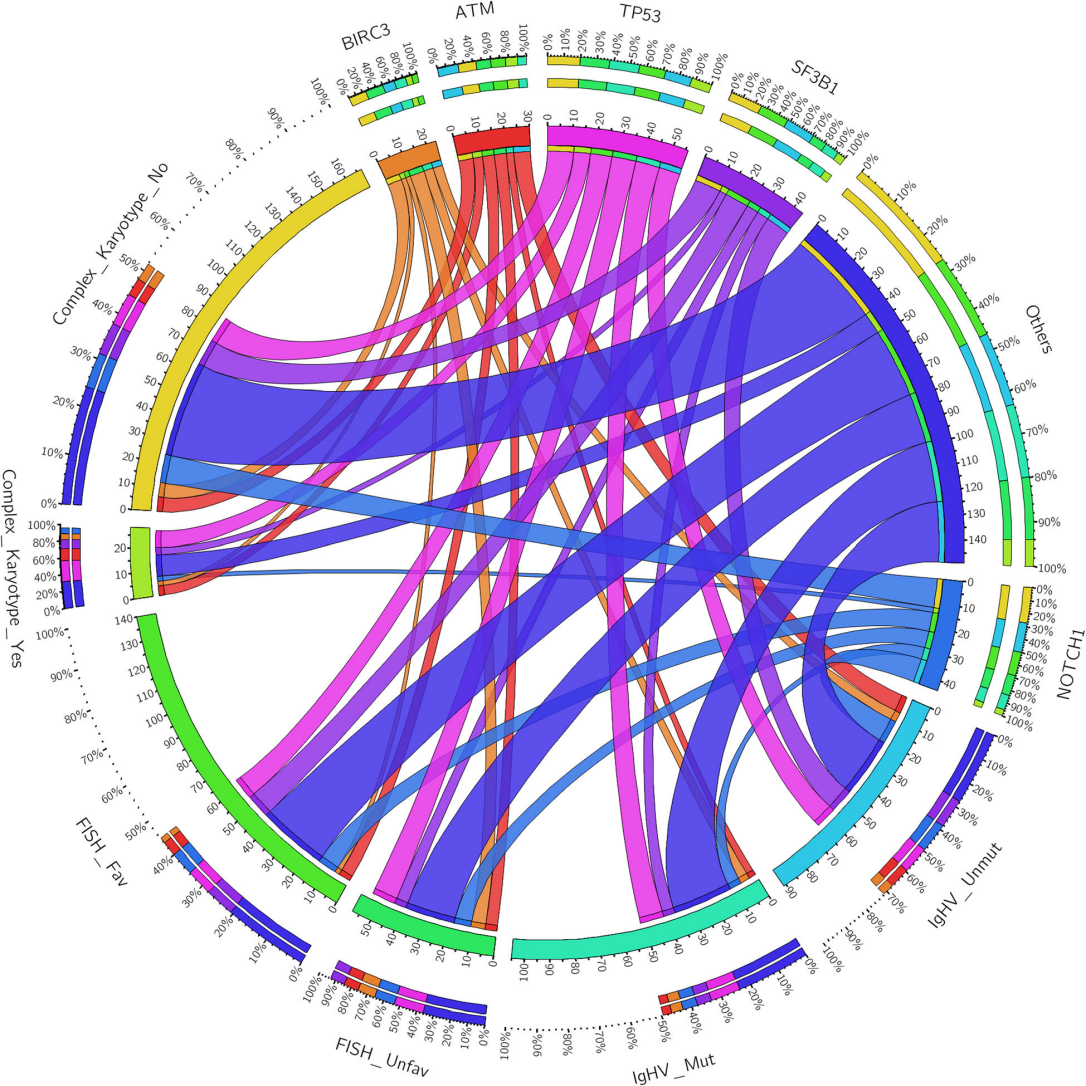
Gene mutations and correlation with genomic features: circos diagrams illustrating pairwise co-occurrence of gene mutations with *IGHV* status, FISH results, and complex karyotype

**Fig. 2:**
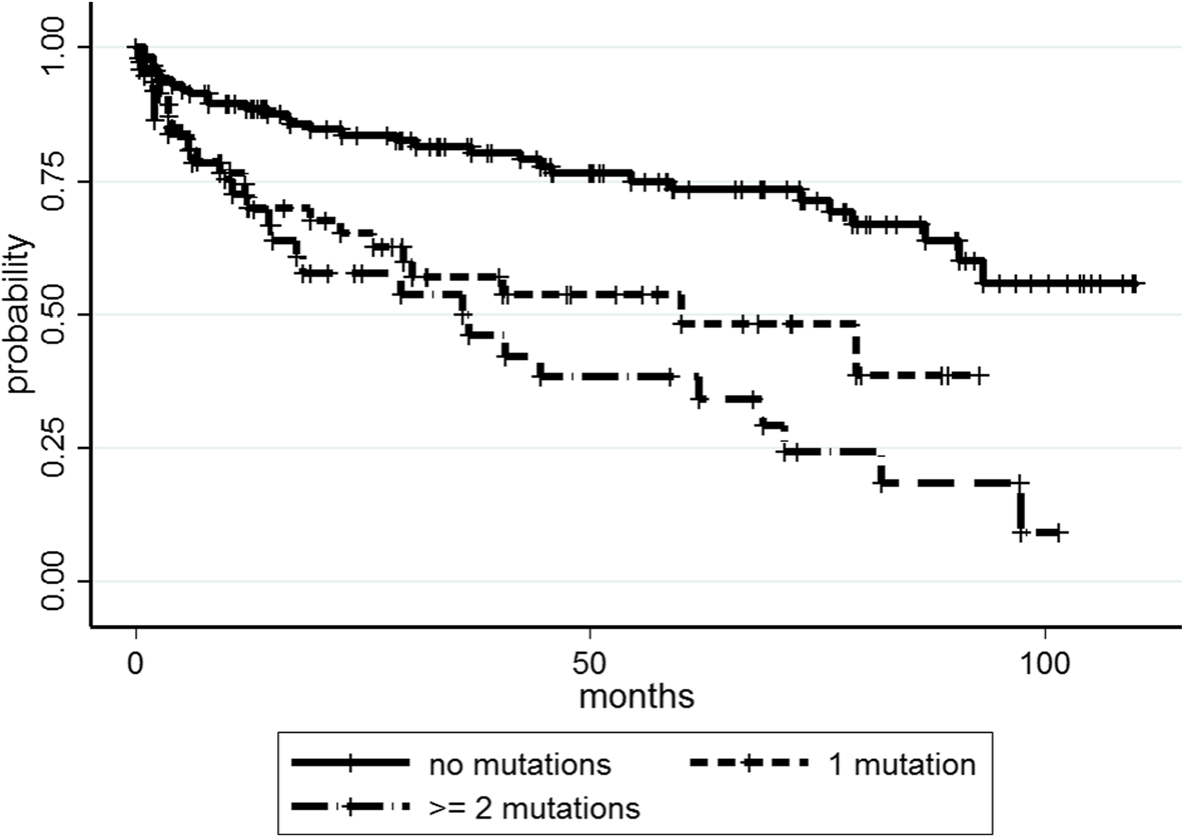
TTFT according to number of mutations by NGS analysis (*p* < 0.001)
