# Supplementary material for: Extensive next-generation sequencing analysis in chronic lymphocytic leukemia at diagnosis: clinical and biological correlations
Source: J Hematol Oncol. 2016 Sep 15;9:88. doi: 10.1186/s13045-016-0320-z (PMC5025606; doi:10.1186/s13045-016-0320-z)
Supplement: Additional file 2: Table S2. — Overview of the mutations observed in the 20 genes. (DOCX 13 kb) [file 13045_2016_320_MOESM2_ESM.docx]

Additional file 2: Table S2. Overview of the mutations observed in the 20 genes.

|  |  |  |
| --- | --- | --- |
| Gene | n. of patients with mutations  Isolated/concurrent | total |
| *TP53* | 6/10 | 16 |
| *NOTCH1* | 6/10 | 16 |
| *SF3B1* | 7/8 | 15 |
| *ATM* | 4/6 | 10 |
| *BIRC3* | 2/6 | 8 |
| *MYD88* | 2/5 | 7 |
| *PTEN* | 3/4 | 7 |
| *FBXW7* | 2/4 | 6 |
| *POT1* | 3/2 | 5 |
| *BRAF* | 1/4 | 5 |
| *ZMYM3* | 2/3 | 5 |
| *KRAS* | 2/2 | 4 |
| *CHD2* | 3/1 | 4 |
| *CDKN2A* | 1/2 | 3 |
| *XPO1* | 1/2 | 3 |
| *NRAS* | 1/2 | 3 |
| *PIK3CA* | 1/2 | 3 |
| *KLHL6* | 1/1 | 2 |
| *DDX3X* | 0/1 | 1 |
| *KIT* | 0/0 | 0 |

Patients are subdivided according to the presence of isolated or concurrent mutations.
