# Supplementary material for: Extensive next-generation sequencing analysis in chronic lymphocytic leukemia at diagnosis: clinical and biological correlations
Source: J Hematol Oncol. 2016 Sep 15;9:88. doi: 10.1186/s13045-016-0320-z (PMC5025606; doi:10.1186/s13045-016-0320-z)
Supplement: Additional file 3: Table S3. — Gene mutations according to the presence of 1 or ≥2 mutations by NGS analysis. (DOC 33 kb) [file 13045_2016_320_MOESM3_ESM.doc]

Additional file 3: Table S3. Gene mutations according to the presence of 1 or ≥2 mutations by NGS analysis

|  | 1 mut | ≥2 mut | P |
| --- | --- | --- | --- |
| *TP53* no/yes | 42/5 | 26/11 | 0.027 |
| *NOTCH1* no/yes | 42/6 | 27/10 | Ns |
| *SF3B1* no/yes | 41/7 | 29/8 | Ns |
| *ATM* no/yes | 44/4 | 31/6 | Ns |
| *BIRC3* no/yes | 46/2 | 31/6 | 0.059 |
| *MYD88* no/yes | 45/2 | 32/5 | Ns |
| *PTEN* no/yes | 44/3 | 33/4 | Ns |
| *FBXW7* no/yes | 45/2 | 33/4 | Ns |
| *POT1* no/yes | 44/3 | 35/2 | NS |
| *BRAF* no/yes | 46/1 | 33/4 | Ns |
| *ZMYM3* | 45/2 | 34/3 | Ns |
| OTHERS no/yes | 39/8 | 24/13 | 0.057 |
